# Cinematic rendering – an alternative to volume rendering for 3D computed tomography imaging

**DOI:** 10.1007/s13244-016-0518-1

**Published:** 2016-09-15

**Authors:** Evelyn Dappa, Kai Higashigaito, Jürgen Fornaro, Sebastian Leschka, Simon Wildermuth, Hatem Alkadhi

**Affiliations:** 1 Institute of Diagnostic and Interventional Radiology, University Hospital Zurich, University of Zurich, Raemistrasse 100, CH- 8091 Zurich, Switzerland; 2 Division of Radiology and Nuclear Medicine, Kantonsspital, St Gallen, Switzerland

**Keywords:** Computed tomography, Image processing, Three-dimensional, Volume rendering, Cinematic rendering

## Abstract

**Abstract:**

Volume rendering (VR) represents today’s standard three-dimensional (3-D) image post-processing technique, and often is used to visualize complex anatomical information. Recently, a novel 3-D technique for post-processing of computed tomography (CT) image data has been introduced, which is called cinematic rendering (CR). The objective of this review is to illustrate the image appearance and potential value of CR in comparison with conventional VR in a number of various applications and different anatomical regions. Similar to VR, CR best visualizes high density and high contrast structures such as bones and contrast-enhanced vessels, but at the same time provides a more natural and photo-realistic illumination of the rendered data. Further research will be necessary for determining possible advantages of CR over conventional VR and over two-dimensional (2-D) image post-processing for CT image data.

***Teaching Points*:**

*• Cinematic rendering is a novel post-processing technique for 3D visualization of CT image data*.

*• Compared to volume rendering, CR results in a more photo-realistic representation of anatomy*.

*• Similar to volume rendering, CR provides best image quality of high density structures*.

## Introduction

Current computed tomography (CT) scanners provide isotropic submillimeter resolution enabling the utilization of various 2-D and 3-D post-processing techniques with excellent image quality. Whereas 2-D techniques such as planar or curved multiplanar reformations (MPR) as well as maximum intensity projection (MIP) images can be considered part of daily radiological routine, 3-D imaging post-processing techniques such as surface shaded display (SSD) and volume rendering (VR) are mainly used to convey complex anatomy. Here, 3-D visualization of CT image data can offer important information compared with 2-D axial images alone both for pre-operative planning and post-treatment follow-up.

While the generation of VR images in the early days was hampered by high computational demands and often was associated with long post-processing times with considerable user interaction, today’s powerful computers allow for a near-real time and automatic rendering of 3-D images in a fast, clinically compatible manner. Thus, VR is today used as the standard technique for the 3-D visualization of CT image data. The main advantages of VR lie in a relatively simple evaluation of different anatomical structures of a larger body region at the same time. Moreover, VR enables the display of CT image data in a coloured fashion [1, 2].

However, the VR technique also suffers from some disadvantages. While VR can be helpful in evaluating complex anatomy, it can also mask anatomical information and, hence, pathology. This has been recently illustrated for the visualization of intracranial aneurysms with VR, where 3-D CT angiography with bone subtraction provided a significantly higher diagnostic accuracy compared to 3-D CT angiography without bone subtraction [3]. Thus, in order to avoid missing critical information, VR images should always be correlated and compared with corresponding MPR images.

Finally, it should be kept in mind that visualization of imaging data with VR – as with all other post-processing techniques such as MIP or SSD—never adds information to the reader beyond what is present in the original source images. It always remains an option for a different representation of imaging data, which might be helpful for anatomically complex structures and disease, and for easier and comprehensible illustration of imaging findings to clinicians.

Recently, a new technique for 3-D visualization of cross-sectional image data from CT has been introduced, which is entitled cinematic rendering (CR). CR works with random sampling computational algorithms and uses different light maps to generate a realistic depiction of medical data. While first studies have started using the CR technique for visualizing anatomy from CT data [4, 5], there is so far—to our knowledge—no publication describing and discussing the new technique in more detail.

Thus, the purpose of this pictorial review is to illustrate the potential value of CR in comparison with conventional VR in various applications and body regions. For doing so, we (*i*) summarized the technical background and potential clinical applications of the conventional VR technique, (*ii*) introduced the technical concept of CR, and (*iii*) selected a number of common and some rare pathology and disease for visualizing the potential of the new 3-D post-processing technique for CT image post-processing.

## Volume rendering – technical background

The technical background of the VR technique has been repetitively explained in detail in a number of previous publications [2, 6]. VR represents a computer algorithm used to transform cross-sectional image datasets (from, e.g., CT or magnetic resonance (MR) imaging) into 3-D images. The VR technique consists of the following two steps: classification of each voxel and image projection. Classification determines how each point on the artificial rays that pass through the data contributes to the pixel value on the picture [7, 8]. For separation of different tissues (e.g., bone, soft tissue), a trapezoid is used for each tissue type. The tissue represented in each voxel of the volume dataset is determined by using predefined attenuation threshold levels and is assigned to a specific colour and opacity. Then, the weighted sum of the percentage of each tissue type represented in the voxel is calculated to determine the overall colour and transparency of each voxel. This step is performed for each voxel and for the whole volume dataset.

Then, the 3-D volume is displayed by using a projection technique. This is done by simulating rays of light, which are projected through the 3-D volume that contains the classified voxels. Each voxel, which is passed by the simulated rays, modulates the colour of the light depending on the assigned colour and transparency and contributes to the final projection and final image.

Finally, 3-D perception in the projected image can be enhanced by implementing additional effects, such as reflections and shadows on the surface of the rendered image. The final image can be manipulated by increasing or decreasing the slope of the trapezoid, which can be observed nearly in real time [8]. When sliding the trapezoid towards lower Hounsfield unit values, more structures with lower attenuation will be included (e.g., smaller vessels), and vice versa.

## Volume rendering – potential clinical applications

### Complex anatomy

Useful clinical applications of the VR technique for visualizing complex anatomy and pathology have been described in many radiological fields and include otorhinolaryngiology [9], neurosurgery and cranio-facial surgery [10], female imaging [1], vascular anomalies and variants [11, 12], and coronary artery anatomy and anomalies [13–15].

### Pre-operative planning

In trauma patients, VR yields information about fragment size and position in relation to adjacent structures [16–19], and can be used as a preoperative and pre-radiotherapy planning tool leading to high satisfaction and acceptability from referring surgeons [20]. Also, imaging in oncology may profit from the application of the 3-D VR technique. VR can help evaluate tumour origin in difficult anatomical settings such as, e.g., in the pelvis [1], and can be used to determine tumour resectability and for preoperative planning [6].

### Post-operative setting

The VR technique can also provide useful information in patients after surgery, such as, for example, for visualizing the results from endovascular aortic repair and the relationship between stent grafts and arterial vessels [21, 22]. An additional application of the VR technique is the visualization of the complex post-operative anatomy after coronary artery bypass graft surgery [23]. Here, the variable course of bypass grafts often crossing the axial image plane several times in different directions might be difficult to appreciate using 2-D post-processing tools alone. In regard to arteries and veins, VR provides visual information, which normally is obtained with conventional catheter angiography. Here, VR is not only noninvasive, but also fast and cost-effective [6].

## Cinematic rendering – technical background

The development of CR was inspired by the entertainment industry based on the quality of computer animation programs in cinema, and had the aim to generate more photo-realistic representations of the human body from CT and MR image data sets.

CR is currently available as a research tool in an open research environment containing various prototype software (Frontier, version 1.0.0, syngo.Via, version VB10A, Siemens Healthcare, Forchheim, Germany) and has so far no approval for clinical use.

The CR technique [24] introduces a new paradigm, enabled by recent advances in computer graphics, to render volumetric medical image data by using a physically based real-time technique [25]. Conventional VR, such as ray casting [26] only considers emission and absorption of energy along a light ray to calculate 3-D images. Scattering effects are modelled using a local gradient shading model. Although simple to compute using a Riemann integral, such conventional approaches neglect complex light paths with multiple scattering patterns as well as light extinction, which might lead to a less artificial and potentially less accurate VR images.

As opposed to conventional VR methods, CR solves the multi-dimensional and non-continuous rendering equation [24] to integrate the light scattered from all possible directions along a ray. Thus, path tracing used in CT integrates a huge number of light rays, each with different paths to form each pixel of the rendered image. Since the number of light paths which can be traced is in theory infinite, and tracing of light paths is computationally expensive, Monte-Carlo simulations are used to generate a randomized subset of light paths with an adequate distribution. The final image is obtained iteratively by progressively averaging numerous Monte Carlo samples representing radiance at random positions with light scattered in random directions.

High dynamic range (HDR) rendering light maps are used for illumination, which lead to a natural illumination of the rendered data, in contrast to synthetic light sources used in conventional rendering methods such as VR [27].

As a result, the physically based VR method called CR computes in real-time the complex physics of lighting effects. It models shadows, ambient occlusion, multi-scattering, and color transmittance as well as sophisticated camera properties, which include concepts such as aperture, exposure and shutter speed. This approach leads to a natural and physically accurate presentation of the medical data, with a focus on an enhanced depth and shape perception [28].

The handling of transparency in CR does not differ from the conventional VR technique. The transparency is computed based on the transfer-function, which assigns colour and opacity to each attenuation value. Potential differences in transparency approaches can be easily adjusted by fine tuning of the transfer-function.

**Fig. 1:**
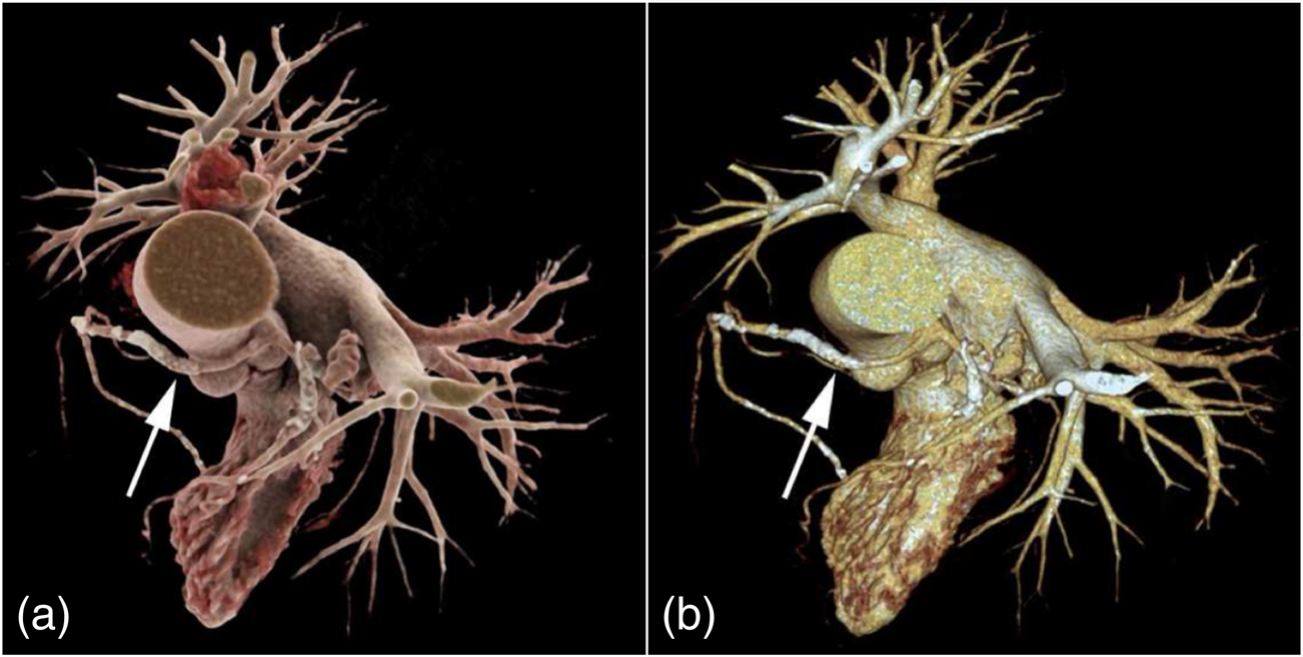
Contrast-enhanced 3-D images of a malignant type coronary anomaly with origin of the right coronary artery (RCA) from the left coronary sinus and interarterial course (*arrows*). Cinematic rendering (CR) (**a**) shows the RCA coursing ventrally to the aortic root to the right side. The volume rendered (VR) image (**b**) depicts the same anomaly from a similar perspective. Note that vessel contour is smoother on CR compared to VR images

**Fig. 2:**
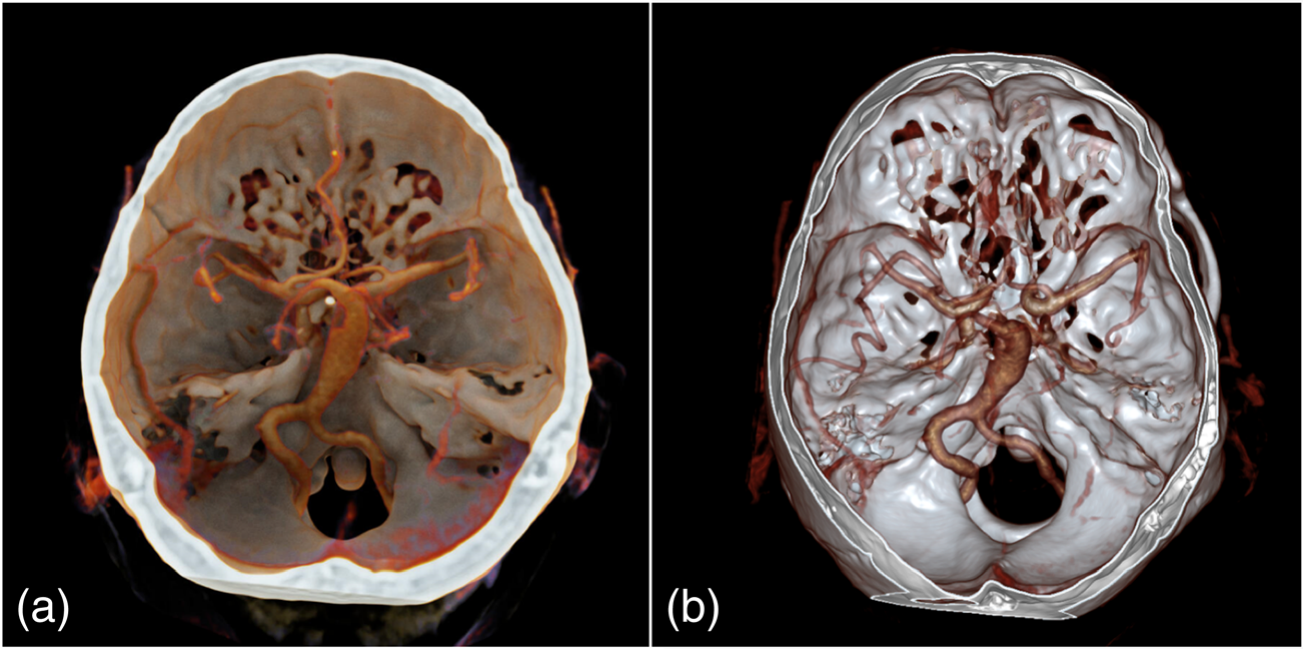
Contrast-enhanced 3-D CT images of a basilar artery aneurysm. CR (**a**) and conventional VR (**b**) depict the base of the skull with the circle of Willis, as well as the fusiform dilation of the basilar artery. Note the more natural representation of the bones and vessels in the CR image

**Fig. 3:**
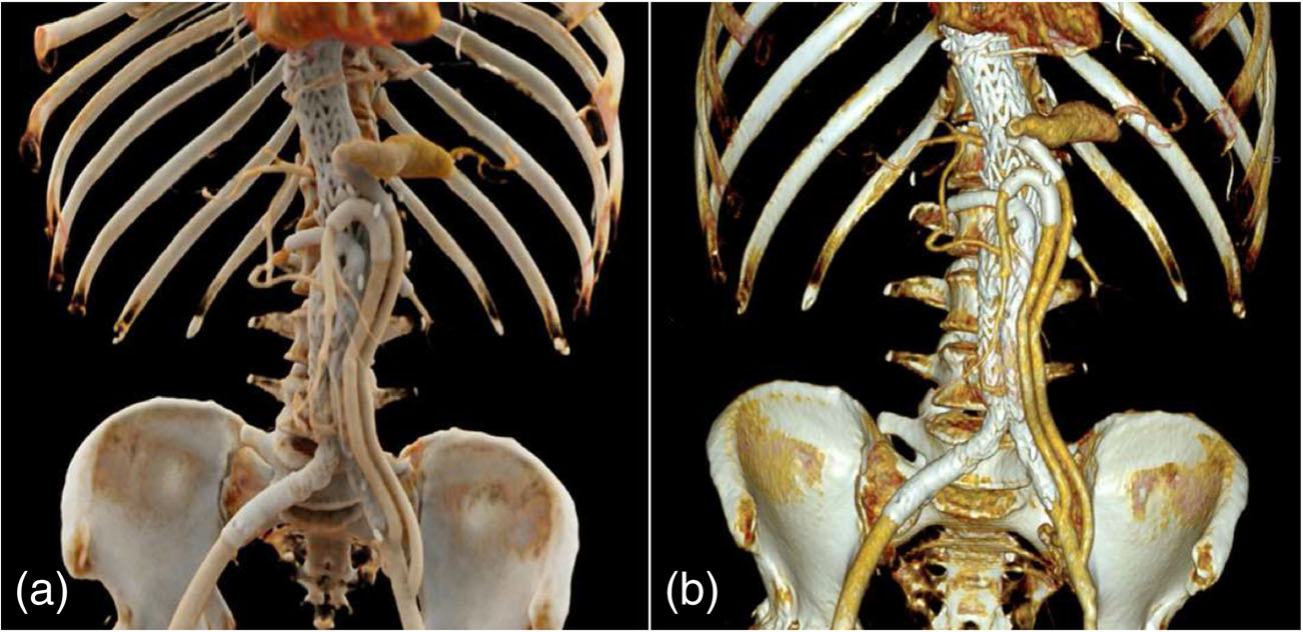
Contrast-enhanced 3-D CT images after endovascular stent graft treatment of the abdominal aorta with visceral debranching in a patient with infrarenal aortic and splenic artery aneurysm, shown with CR (**a**) and with VR (**b**)

**Fig. 4:**
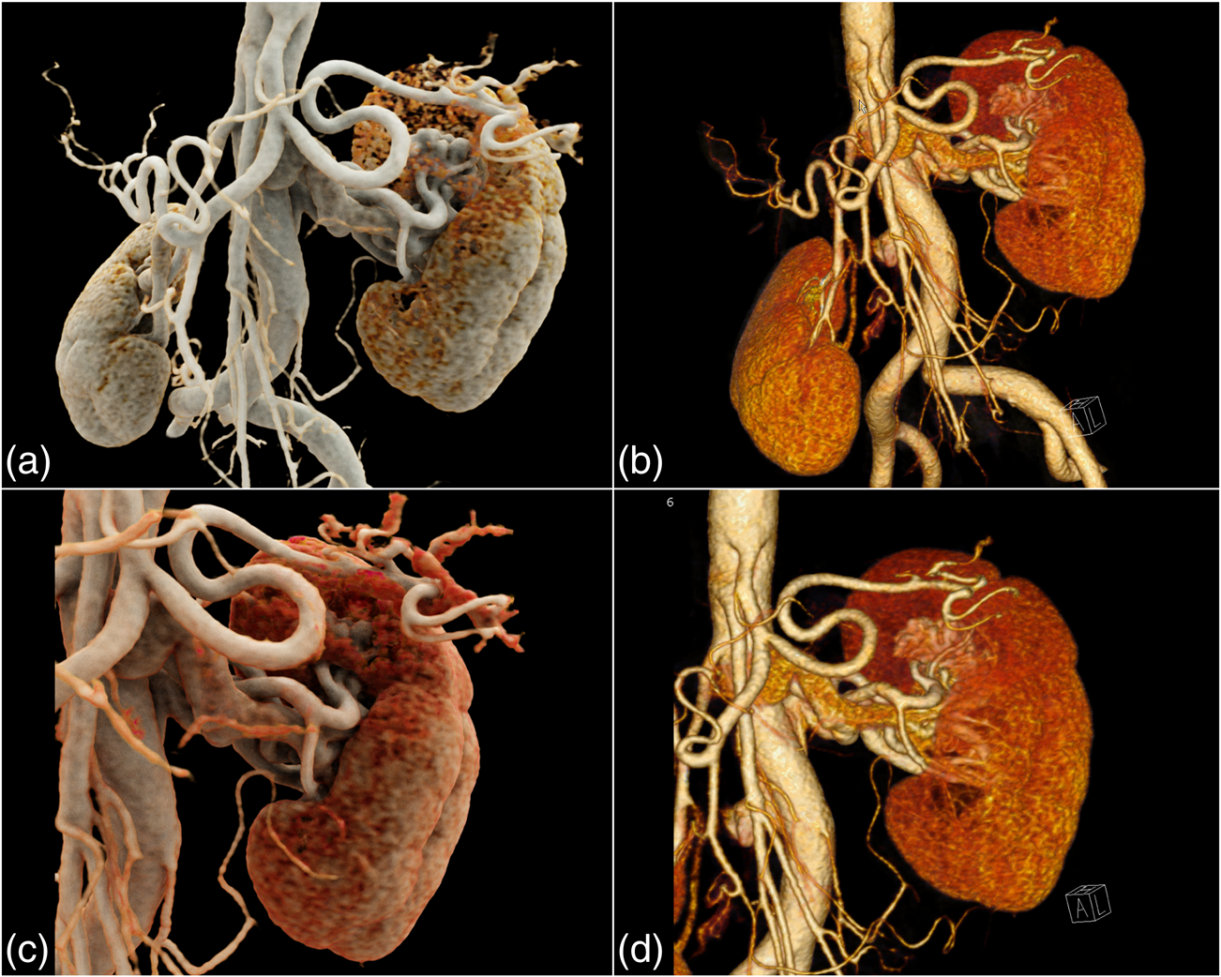
Contrast-enhanced 3-D CT images of an arteriovenous malformation (AVM) in the upper pole of the left kidney. CR (**a**) and VR (**b**) images and close-up view (**c**, **d**) images allow for a more detailed depiction of the AVM. The tortuous vessels at the upper kidney pole are visualized with a more realistic representation on CR than on VR images

**Fig. 5:**
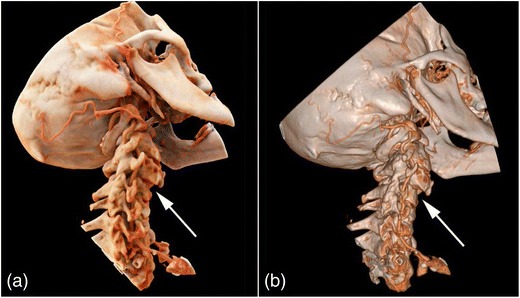
Contrast-enhanced 3-D CT images of a cervical spine injury with luxation of C3/4. Both CR (**a**) and VR (**b**) images show the anterior subluxation of the cervical vertebrae (arrows). The enhanced lightning of CR creates a high contrast between vessels and bone, making it easy to follow the course of the vertebral artery

**Fig. 6:**
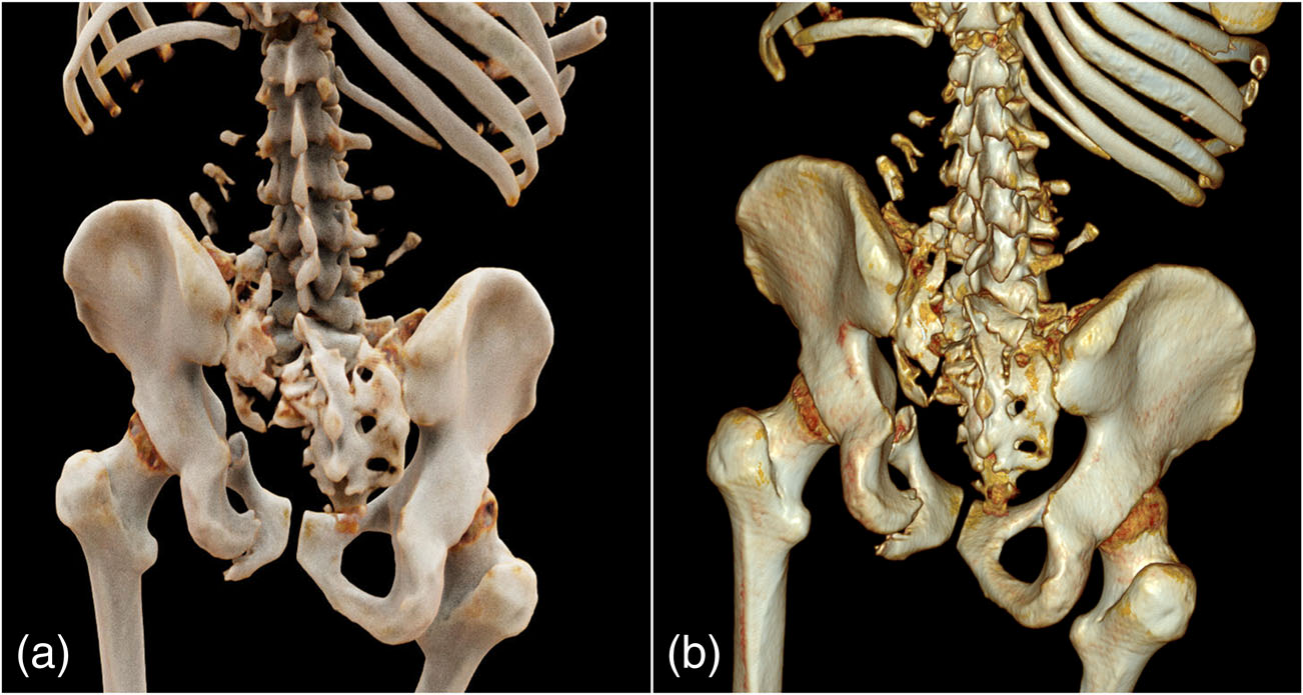
Non-enhanced 3-D CT images of multiple traumatic fractures of the spine and pelvis. Both CR (**a**) and VR (**b**) demonstrate multiple fractures of the ribs, lumbal transverse processes, sacrum, and pubic bones

**Fig. 7:**
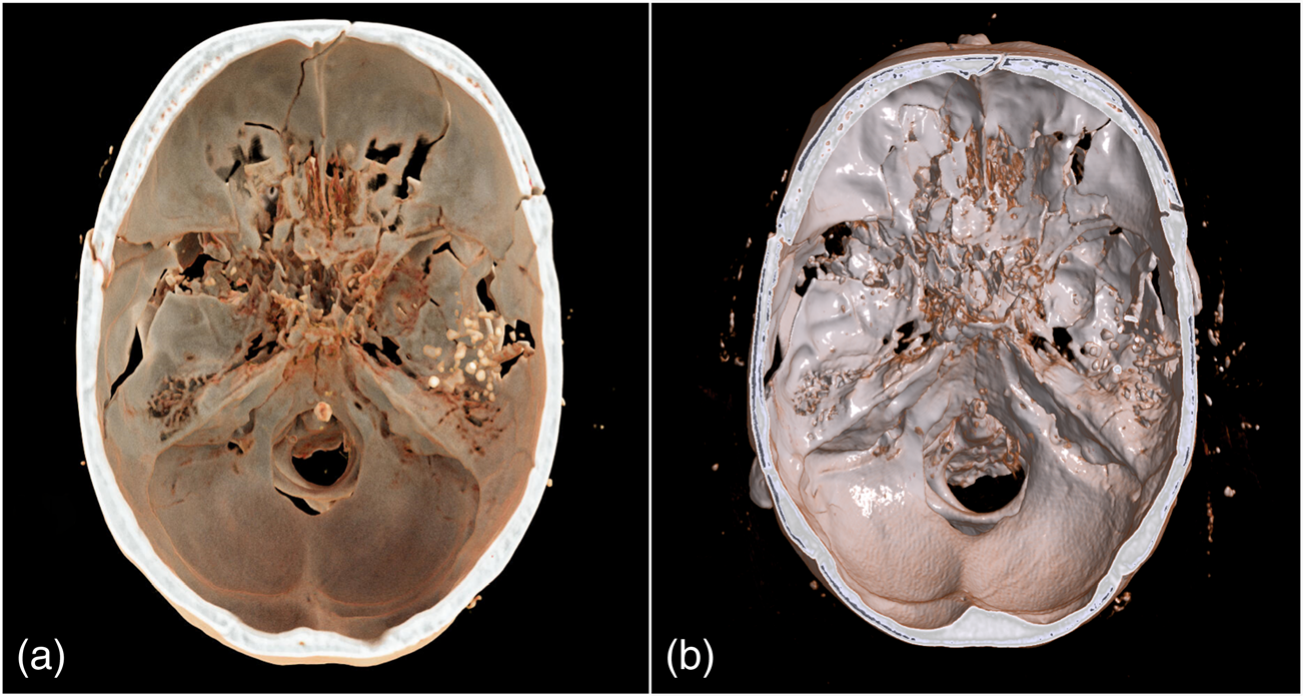
Non-enhanced 3-D CT images of a head shot injury. Both CR (**a**) and VR (**b**) show the bullets and extensive damage of the skull base. The more photo-realistic representation of anatomy with CR allows for an easy identification of the complex fracture course

**Fig. 8:**
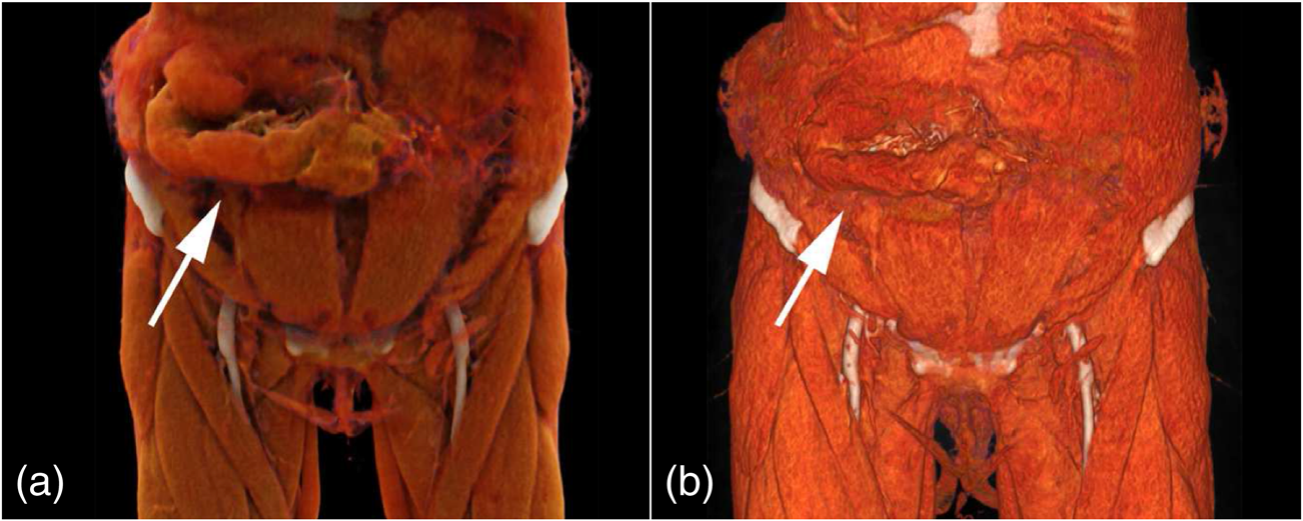
Non-enhanced 3-D CT images of traumatic herniation of small bowel through the anterior abdominal wall. CR (**a**) and VR (**b**) show the soft tissues of the abdominal wall and upper thigh along with herniating of the bowel loops (*arrows*). CR allows for a natural representation of anatomy

**Fig. 9:**
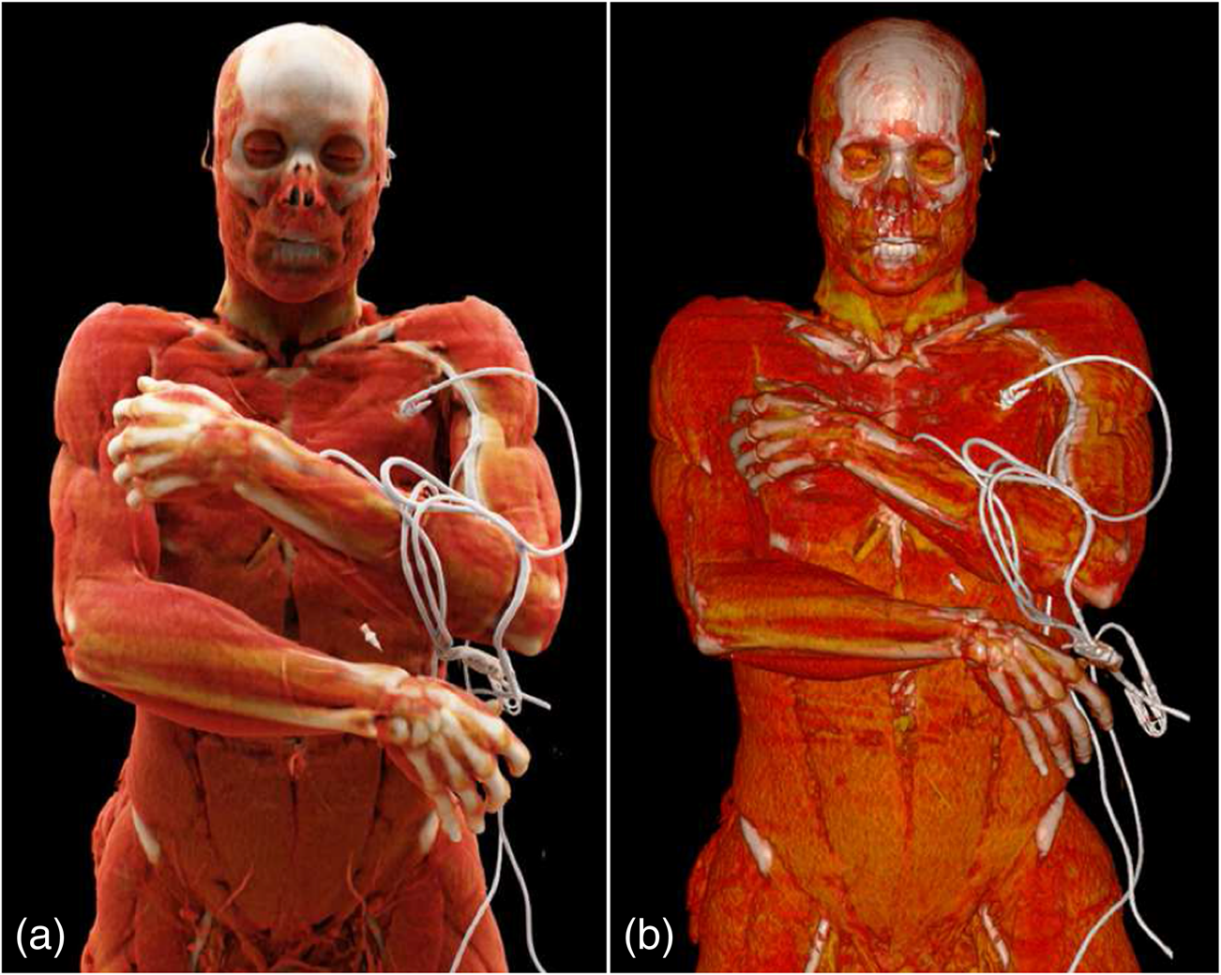
Contrast-enhanced 3-D CT images of a patient with polytrauma. CR (**a**) and VR (**b**) visualize the soft tissues of the head, neck, chest and abdomen. Note the differences in photo-realistic representation of 3-D anatomy in CR as compared to VR images

Representative image examples of CR in comparison with VR are provided in Figs. 1, 2, 3, and 4 for vascular anatomy and pathology, in Figs. 5, 6, and 7 for bones and skeletal disease, and in Figs. 8 and 9 for visualization of soft tissue anatomy. All CT source images were reconstructed with our default settings using a slice thickness of 2 mm (increment 1.5 mm), except for coronary CT angiography, which was reconstructed with a slice thickness of 0.75 mm (increment 0.5 mm). All CT angiography data were acquired after the intravenous injection of 50–80 ml non-ionic, iodinated contrast media (concentration 300–370 mg J/ml, depending on the body region).

## Discussion

Both, VR and CR have the same general rendering concept in common: segmentation of data based on voxel attenuation and use of colour look-up tables taking into account opacity and brightness. As a consequence, both VR and CR share also the same problem of potentially masking findings in datasets by inappropriate use of rendering parameters and by adjacent structures [3]. Albeit CR currently is only provided by a single vendor in a pre-clinical research tool with still limited display options, CR can provide the same advanced display functions such as VR including flying-through, flying-around and multiple views of the volume data with independent parameters in equal segments of the displayed window [2, 6].

Due to the abovementioned similarities between CR and VR, there are no major differences in the visualization of CT image data when regarding the diagnostic value of the presentation. Similar to VR, we found that particularly high-contrast structures such as contrast-enhanced vessels and bones can be depicted with high quality also with CR. At the same time, we found a major improvement of CR for the perception of depth and soft tissue structures providing a more photo-realistic depiction of human anatomy and disease.

In general, lighting in 3-D rendered images such as VR and CR is a function of ambient, diffuse, and specular light properties. In both techniques, the brightness of each voxel is defined by the distribution of these light properties, which results in different lighting of different body parts relative to the artificial light source introduced into the volume, giving rise to 3-D impressions of the images. In VR, the differences in light emitted to the voxels are rather small. In contrast, CR uses a more complex lighting model taking into account the effect of lighting for other voxels and subsequent reflections as well. Also, the effect of body parts blocking the trace from the artificial light source to other structures introduces shadowing into the images. As a result of the differences in lighting functions—as being illustrated in the representative image examples of this pictorial review—CR images go along with a more natural image impression as compared to conventional VR.

Despite the potential benefits of CR compared to VR in the visualization of volume datasets, there is a higher computational power demand required for the CR technique because of the more complex lighting model. Therefore, real-time display of, for instance, rotating the CR image is currently interrupted by repetitive recalculation processes. Rendering of the final image needs some seconds, ranging from 5 to 30 s depending on the quality of the resulting image [28].

One major limitation of this pictorial review is that we focused our comparison of VR and CR on some clinical cases to highlight the differences in image appearance between both post-processing methods. However, we did not perform an analysis of both methods regarding the diagnostic performance for the diagnosis of specific pathological entities. Here, future studies should determine whether or not there is an improvement of the new 3-D imaging technique described herein.

## Conclusion

This pictorial review aimed at a first, preliminary demonstration of a new post-processing technique for the 3-D visualization of CT image data. Our initial experience indicates that CR of CT images is particularly impressive when high density and high contrast structures such as bones and contrast-enhanced vessels are to be visualized. The main innovation as compared to conventional VR appear to be the more natural and photo-realistic representation of the CT image data, with an enhanced and more natural depth and shape perception. Future studies should aim at determining whether the CR technique offers advantages over cross-sectional and 3-D CT images in terms of a diagnostic gain.
